# Numerical analysis of underground tunnel deformation: a case study of Midroc Lega-Dembi gold mine

**DOI:** 10.1038/s41598-024-57621-x

**Published:** 2024-04-04

**Authors:** Nagessa Zerihun Jilo, Siraj Mulugeta Assefa, Eleyas Assefa

**Affiliations:** Department of Civil Engineering, College of Engineering, Addis Ababa Science and Technology University, Addis Ababa, Ethiopia

**Keywords:** Deformation, Geotechnical parameters, Mining tunnel, Numerical method, Rock bolt support, Civil engineering, Natural hazards

## Abstract

Undertakings in underground mining are often complicated, particularly in situations where geotechnical conditions are not favorable. This study investigates the collapse of tunnels at the Lega-Dembi gold mine in Southern Ethiopia, an area characterized by weak talc formations. The persistent deformation of tunnels poses a threat to the safety of workers and mining operations. In this study, a numerical method that combines continuum and discontinuum approaches is employed to analyze tunnel failures. Additionally, the study evaluates the effect of geotechnical parameters on tunnel deformation, considering various support systems. The results indicate that a combination of rock bolts and shotcrete is effective in mitigating tunnel deformation. Furthermore, the study identifies the geological strength index and unconfined compressive strength as the most influential parameters on tunnel deformation. The findings also suggest appropriate support systems for managing underground instability and enhancing safety measures in weak geological formations.

## Introduction

Tunnels have various applications in civil and mining engineering, such as in power generation, transportation, drainage, and irrigation. In underground mining, tunnels allow access to the ore body and facilitate the transport of the mined material. The stability of these tunnels is crucial for profitability and efficiency of mining operations. Hence, it is important to comprehend the rock mass behavior during and after tunneling, which can assist in designing or modifying the support and excavation methods and avoiding possible failures near openings.

The complex geology and high geostress of rock masses pose several challenges for tunnel construction and operation in underground mining^1–3^. Rock strength is greatly affected by discontinuities such as shear zones, dykes, faults, joints, and bedding planes^4–6^. The structural features of the rock mass and the balance between rock strength and stress influence the stability of a tunnel^7^. Moreover, the in-situ stress can cause large failures and deformations in the surrounding rock during the maintenance and installation of the tunnel support system^8,9^. Therefore, tunnel safety is critical for underground mining.

The behavior of rock masses around tunnels in underground engineering, particularly in complex geological formations, has been widely studied using numerical simulation methods. These methods can be classified as either continuum or discontinuous methods. The finite element method (FEM) can handle material heterogeneity, complex boundary conditions, and in-situ stresses in rock mechanics problems^10^. The FEM has been used to study the performance of underground tunnels. For example, Abdellah et al.^11^ used 2D FEM to examine the effects of certain parameters on tunnel stability. Sebbeh-Newton et al.^12^ showed the importance of 2D FEM in realistic support design for tunneling. Karakus and Demirci^13^, Do et al.^14^, and Janin et al.^15^ used the 2D FEM to predict the rock mass response under different excavation cycles. However, 2D FEM requires an approach that can account for the 3D tunneling effect of the relaxation process^14,15^.

The finite difference method (FDM) can address the nonlinear behavior of rock materials. Some applications of FLAC include estimating the subsidence due to coal seam exploitation; Alejano et al.^16^, studied the effect of different factors on the roof settlement of a tunnel intersection, proposed supporting strategies Hsiao et al.^17^, and predicted the surface subsidence of a coal mine using a three-dimensional model^18^. FLAC is suitable for simulating the nonlinear behavior of rock masses^19^.

The boundary element method (BEM) is a numerical technique that solves boundary integral equations by discretizing the boundary, approximating the solution functions using shape functions, incorporating boundary conditions, and estimating the displacements and stresses in the domain^20^. The BEM is more accurate than the FDM and FEM because it directly solves the integral equations directly^21^. BEM can handle very large domains because it discretizes only the boundary. Some applications of BEM involve solving anisotropic half-plane problems, studying the effect of material anisotropy on stress distributions^20^, simulating rock joints and faults by dividing the domain into boundary element regions and assigning joint behavior for the interfaces^22^, and analyzing the mechanical behavior of a twin tunnel in multi-layered formations^23^.

The Distinct Element Method (DEM) is a valuable tool for assessing the stability of tunnels and underground structures. In DEM, the rock mass is represented by individual blocks with defined boundaries. Discontinuities that occur during underground excavation can influence rock failure. The 3D DEM considers both the 3D effects of tunneling and the discontinuous nature of rock masses. Researchers, such as Kulatilake et al.^24^, Shreedharan and Kulatilake^25^, and Xing et al.^26^ used 3DEC software to investigate the stability of tunnels in coal mines in China and the United States. The 3D DEM has proven to be a reliable method for analyzing the stability of underground excavations.

The purpose of this study is to assess the stability of rock masses near tunnels in the Midroc Lega-Dembi underground gold mine, which is one of the largest gold mines in Ethiopia. The mine has a projected lifespan of 10–25 years, depending on its current production rates; however, it faces considerable geotechnical challenges owing to the complex and diverse geological composition of the region. Development drifts used to access and transport the ore often encounter areas of low rock mass quality or large fault zones, which can cause significant deformations and failures in the rock masses. These failures are more evident at tunnel junctions and in areas of poor rock mass quality where the stress concentration and rock mass weakness are high. Therefore, ensuring the safety and stability of these tunnels is vital for mine development, and the selection of a suitable tunnel support system is necessary. This study intends to provide a comprehensive geotechnical investigation and analysis of rock masses around tunnels, and to recommend optimal tunnel support systems based on rock mass classification and numerical modeling using RS2, FLAC3D, and 3DEC.

### Description of the case study

#### Location

**Figure 1:**
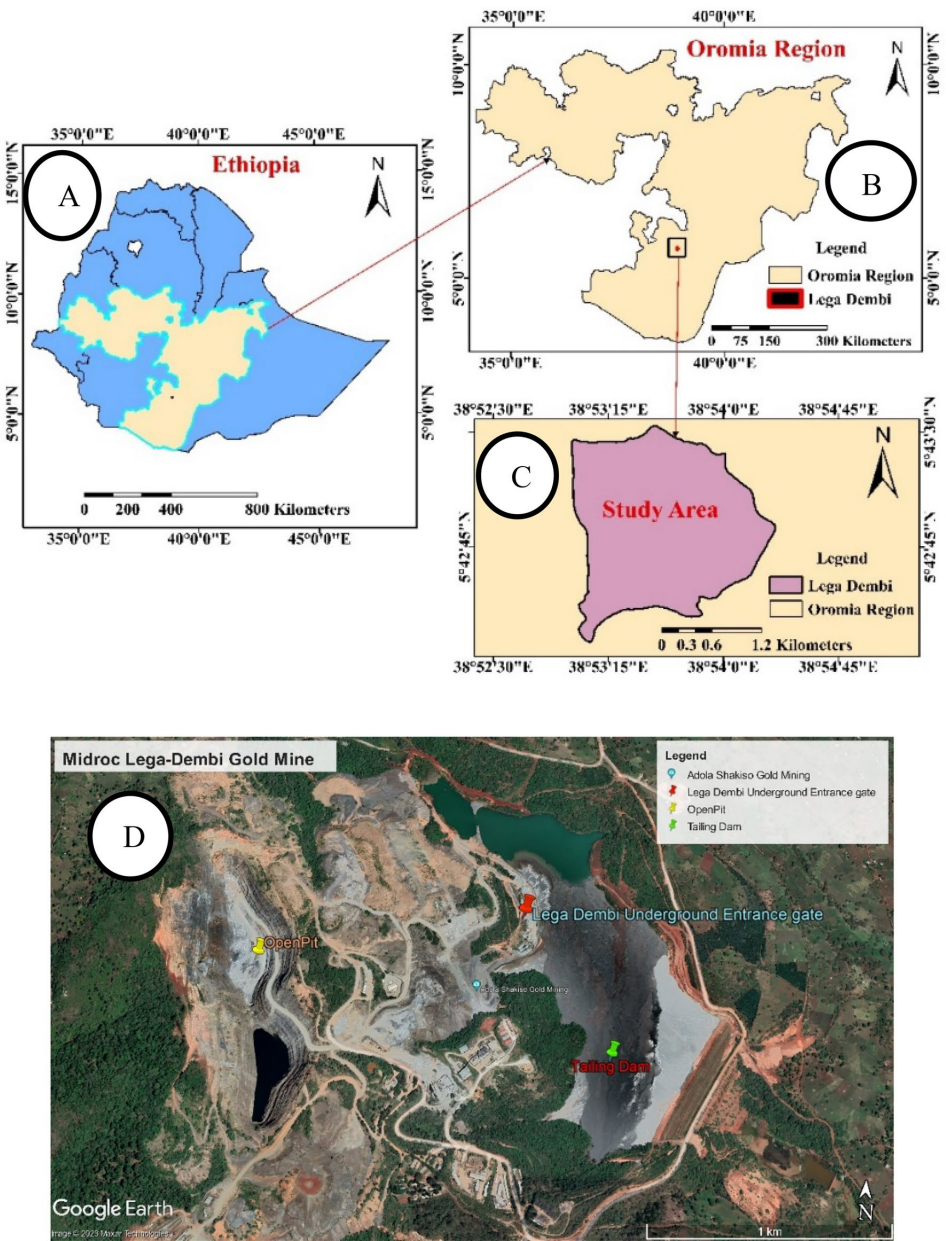
Map of the study area (**a**–**c**) created using ESRI ArcGIS, ArcMap 10.1. Source: Esri, DigitalGlobe, GeoEye, Earthstar Geographics, CNES/Airbus DS, USDA, AEX, Getmapping, Aerogrid, IGN, IGP, swisstopo, and the GIS User Community. and (**d**) https://earth.google.com/web/@5.71507645,38.89306644,1854.90749504a,2685.80779062d,35y,-0.05030178h,57.1959916t,0.00789995r/data=OgMKATA.

This study investigated the collapse of a mining tunnel under the Lega-Dembi Mountains, which is approximately 440 m deep. The tunnel, which belongs to the Midroc Lega-Dembi gold mines in southern Ethiopia, traverses weathered rocks. These mines are the country’s most prolific gold sources and are located near other rich deposits of gold and base metals in the Adola and Magado belts. The project site in the southern Oromia region covered 144 square kilometers and is approximately 550 km from the capital, Addis Ababa. Its coordinates are 5°42′00″–5°44′00″ N and 38°52′30″–38°54′30″ E, and its highest elevation is around 2200 m (Google Earth). (Fig. 1) depicts a map of the site and its surroundings.

#### Regional geology

**Figure 2:**
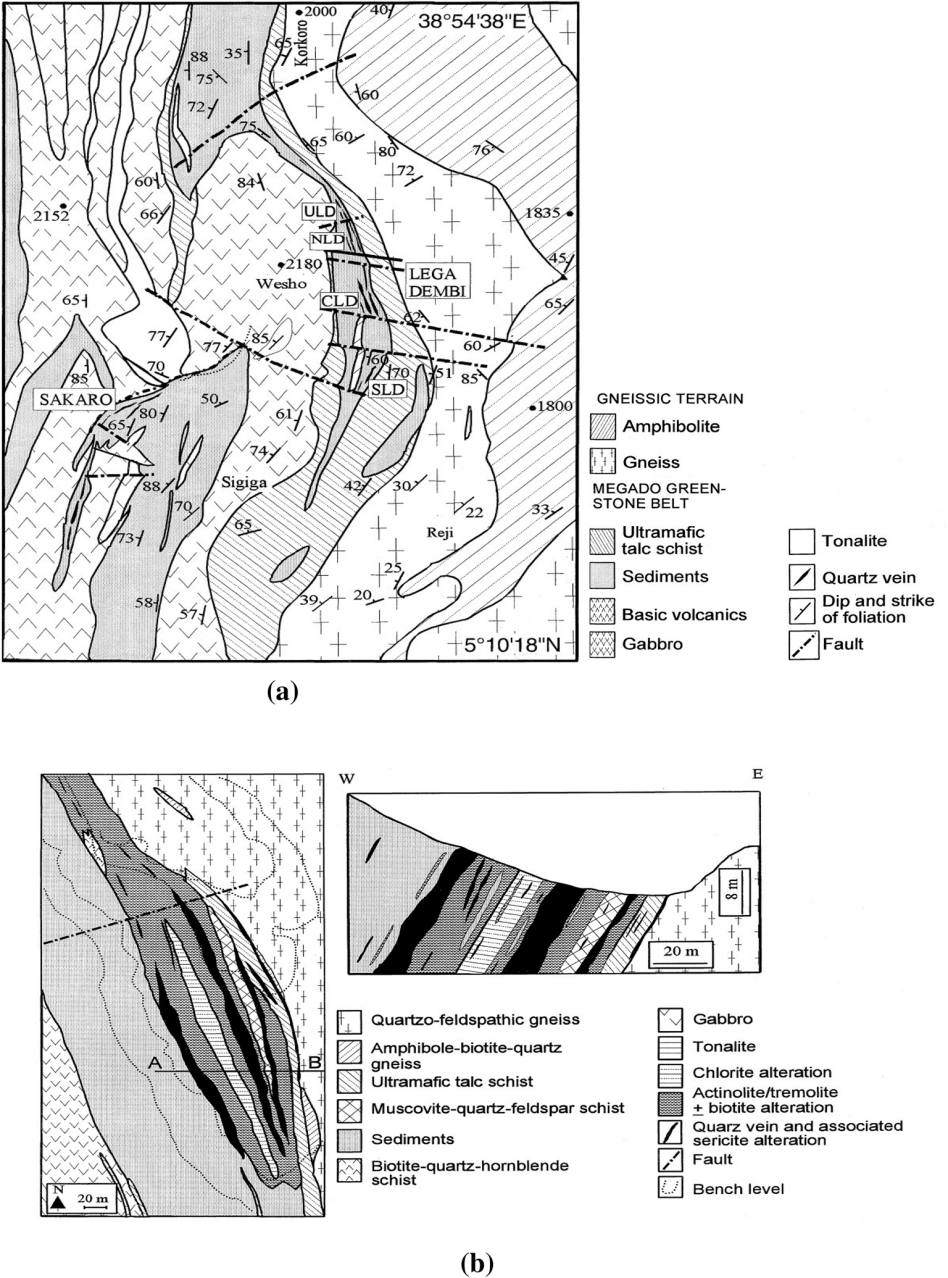
Regional geology of Lega-Dembi area (**a**) and Lega-Dembi underground mine geology study section of the site after Billay et al.^27^ (**b**).

This study investigated the complex tectonic evolution of the Adola Belt in southern Ethiopia. This belt is comprised of greenstone belts with volcanic and sedimentary rocks. The belt was affected by various shear zones with different kinematics and orientations. The Adola Belt has shear zones and fold systems that trend north–south, parallel to the orogen, and oblique strike-slip shear zones that trend NW–SE^27,28^, as depicted in Fig. 2a. The belt also has diverse geological structures such as shear zones, joints, faults, folds, and foliations^29^. The faults in the study area are predominantly normal dip-slip faults, which indicate extensional tectonics during the Late Precambrian. The fault planes are generally steeply inclined, ranging from 60° to 90°, and the throws vary from a few metres to several tens of metres. The faults govern the distribution and orientation of gold-bearing quartz veins, which follow N–S trending fault zones. The study area is situated on the eastern limb of the Lega-Dembi graben-syncline, a large-scale structure that controls gold (Au) mineralization. The Lega-Dembi mine is the largest gold producer in Ethiopia, producing approximately 4500 kg of gold annually. The mine has a total deposit of approximately 37,694,766 tons of gold, implying an average grade of approximately 3.6 g/t^3^. However, the grade may differ significantly along the strike and depth of the orebody depending on the degree of hydrothermal alternation and quartz-veining. The Lega-Dembi gold deposit is near the contact between quartz-feldspathic gneisses and metasediments along the Lega-Dembi-Aflata shear zone. The study area has three sets of faults in different directions: north–south, northeast, and northwest. Geological investigation along the tunnel alignment revealed six rock formations. These include gneiss-intercalated amphibolite schists, quartz-feldspathic mica schists, biotite actinolite schists, quartz veins, talcose biotite actinolite schists and carbonaceous quartz mica schists (Fig. 2b). These rock formations have different characteristics and properties that affect the tunnel performance and stability.

#### Site observation

**Figure 3:**
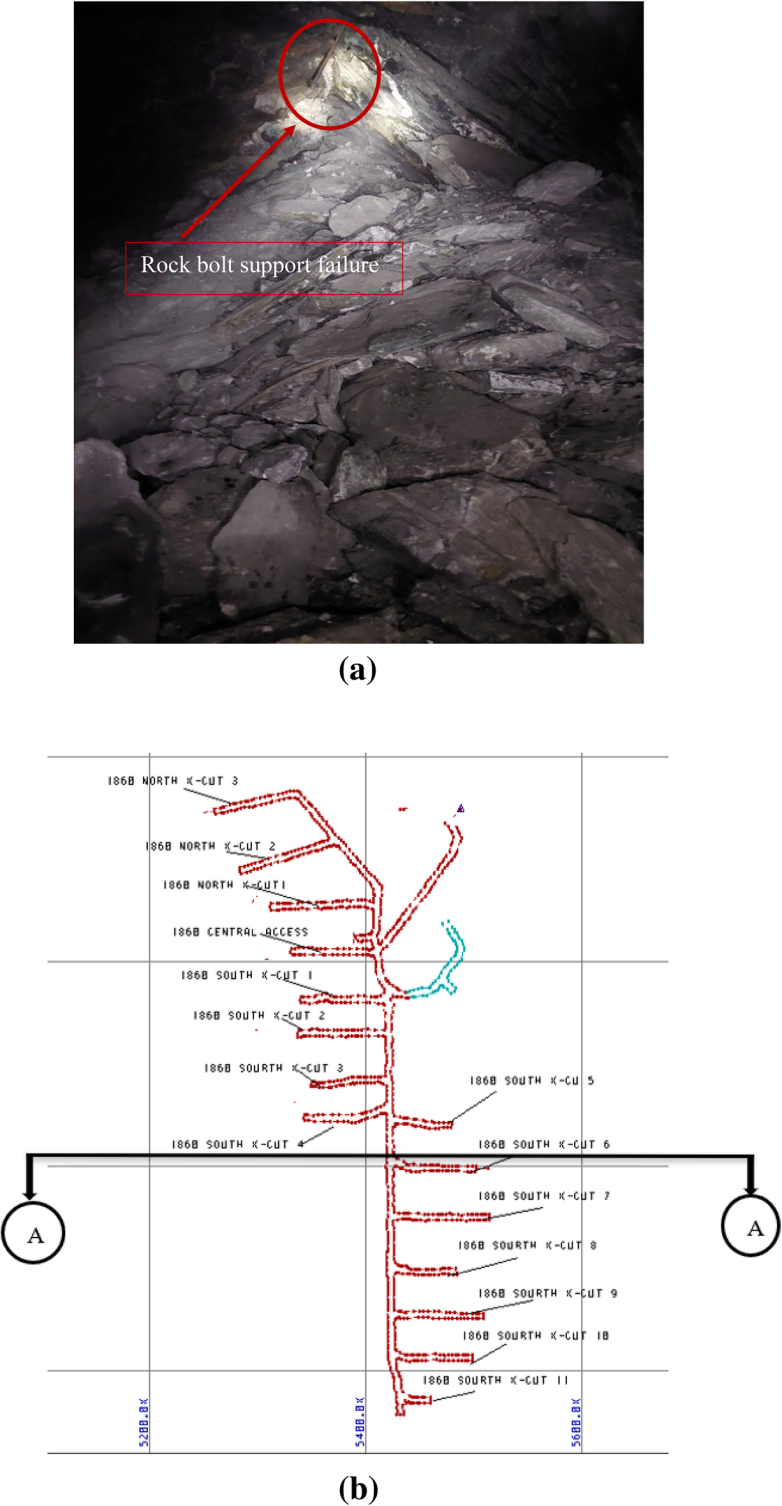
Failed tunnel section (**a**) and section A-A plan view (**b**).

The Midroc Lega-Dembi underground mining tunnel, located in Ethiopia, East Africa, is a challenging project that has undergone three major failures since 2018. The first failure occurred in 2018 when the tunnel face collapsed because of rock instability. This compromised the structural integrity of the underground facility and necessitated the installation of swellex rock bolts to reinforce rock mass. Swellex rock bolts are steel tubes that are hydraulically expanded under high water pressure, thereby creating a strong bond with the rock. The swellex rock bolts had a length of 4 m, which was adequate for providing sufficient support and stability to the structure^30^. The second failure in 2019 resulted in damage to approximately 20 m of the headrace tunnel, rendering it unusable. The third failure in January 2021 was caused by massive rockfall, as shown in Fig. 3a. The figure also shows the failure of rock bolts that were implemented as mitigation measures. The rock bolts failed to prevent the collapse of the large rock mass, which occurred for the third time during this period. The locations of tunnel failures at the mining site are delineated in sections A-A in the mining plan view. This section corresponds to Southern Crosscut 3, as illustrated in Fig. 3b. This failure poses a significant challenge for companies and the gold-mining industry. The headrace tunnel has a length of 1.3 km and is one of the most difficult to construct and maintain. The rock mass failure exhibited elastoplastic behavior with a long squeezing deformation. This indicates that the failure event was associated with a weak rock mass strength and viscous plastic deformation. The analysis focused on the location of the current failure, Lens-2. The tunnel failure and cross-sectional area of the study area are shown in Fig. 3.

## Materials and methods

### Data selection

The numerical analysis used the mechanical properties of the rock mass obtained from the laboratory tests and empirical correlations. The strength parameters of the host rock, such as the Young’s modulus, Poisson’s ratio, cohesion, and friction angle, were used to perform the simulation. These parameters were determined through laboratory tests of the rock samples collected from the site. Rock samples were obtained from drill cores of boreholes drilled along tunnel alignment. The test results were used to calculate the strength parameters of the rock by using empirical and analytical formulas. The horizontal stress in the ground is an important factor that affects the stability of the tunnel excavation. The horizontal stress depends on the overburden depth, rock-mass quality, and tectonic history of the region. In this study, the horizontal stress was estimated by using Eq. (1) proposed by Hoek E. (2002), which is based on the coefficient of earth pressure at rest, Ko′. The formula is:

1 $$\sigma_{H} = k_{0}^{1} \sigma_{\nu } = k_{0}^{\prime } \rho_{d} gZ$$

where σ_H_ is the horizontal stress, σv is the vertical stress, Ko′ is the coefficient of earth pressure at rest, ρ is the density of the rock, d is the overburden depth, g is gravitational acceleration, and z is the vertical coordinate. The coefficient of earth pressure at rest, Ko′, was determined by using the empirical correlation with the rock mass rating (RMR) given by (Hoek E. 2002), The RMR was evaluated by using the geological data and the borehole logs from the site.

Table 1 presents the empirical correlations and their references. Table 2 summarizes the joint properties derived from the field and laboratory tests. Rocdata software from Rocscience (2002) was used to compute the Hoek–Brown parameters from the rock mass properties using Eqs. (2)–(4). The obtained parameters are listed in Table 3.

**Table 1:** Rock mass properties^31^.

Block properties	Unit	Value
Rock mass rating (RMR)	–	IV
Unconfined compressive strength (UCS)	MPa	37
Geological strength index (GSI)	–	32
Material constant (mi)	-	12
Young’s modulus (E_rm_)	MPa	1241.1
Density (γ)	kN/m^3^	26.8
Angle of internal friction (φ)	[^o^]	23.5
Cohesion (C)	MPa	1.85
Bulk modulus (K)	MPa	1011.75
Shear modulus (G)	MPa	466.96
Poisson’s ratio (ν)	–	0.3
Lateral stress ratio (k_o_)	–	0.45

**Table 2:** Joint properties (estimated from raw data).

Joint properties	Unit	Value
Joint normal stiffness (J_kn_)		1.2761
Joint shear stiffness (J_ks_)	MPa	0.550
Joint angle of internal friction (φj)	[^o^]	30
Joint compressive strength (C)	Kg/cm^2^	1066
Joint roughness coefficient (JRC)	–	2
Dip and Dip direction (D/DD)	[^o^]	265/60
Effective normal stress	kg/cm^2^	0.02

**Table 3:** Hoek–Brown constant (obtained from Rocdata software).

Hoek–Brown classification		Hoek–Brown criterion		Rock mass parameters		Unit
Sigci	37 MPa	mb	0.471	Sigt	− 0.009	MPa
GSI	32	s	0.0001	Sigc	0.333	MPa
mi	12	a	0.520	Sigcm	3.067	MPa
D	0.5			Em	1618.68	MPa

Equation (2) Reduced value of a material constant

2 $${{{\rm mb} = {\rm mi} \times {\rm exp} }}\frac{{\left( {\text{GSI - 100}} \right)}}{{\left( {\text{28 - 4D}} \right)}}$$

Equation (3) Hoek–Brown material constant (s)

3 $${\text{s = exp}}\left( {\frac{{\left( {\text{GSI - 100}} \right)}}{{\left( {\text{9 - 3D}} \right)}}} \right)$$

Equation (4) Hoek–Brown material constant (a)

4 $${\text{a}}\; = \;\frac{{\text{1}}}{{\text{2}}}{\text{ + }}\frac{{\text{1}}}{{\text{6}}}\;\left( {\left( {{\text{e}}\;{\raise0.7ex\hbox{${ - {\text{GSI}}}$} \!\mathord{\left/ {\vphantom {{ - {\text{GSI}}} {{\text{15}}}}}\right.\kern-\nulldelimiterspace} \!\lower0.7ex\hbox{${{\text{15}}}$}}} \right) - \left( {{\text{e}}\;{\raise0.7ex\hbox{${ - {\text{20}}}$} \!\mathord{\left/ {\vphantom {{ - {\text{20}}} {\text{3}}}}\right.\kern-\nulldelimiterspace} \!\lower0.7ex\hbox{${\text{3}}$}}} \right)} \right)$$

### Numerical analysis

**Figure 4:**
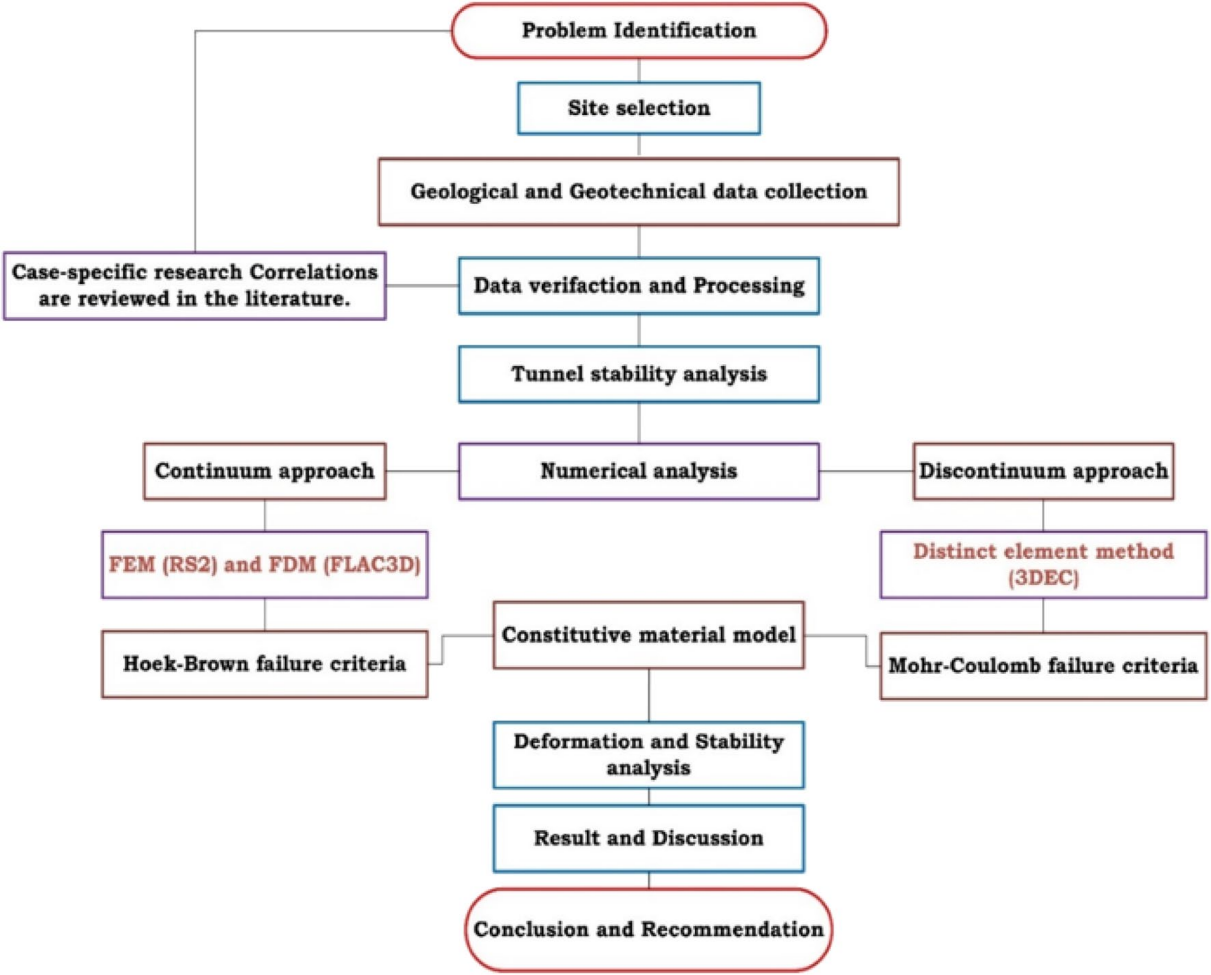
General methodological flowchart of the study.

The objective of this study is to examine the deformation of mining tunnels using numerical simulations. Two types of models were developed: a 3D model using FLAC3D and 3DEC software and a 2D model using RS2 software. Several simulations were conducted with varying parameters and conditions, and the results were analyzed. Figure 4 illustrates the research methodology employed in this study.

#### Geometric model

**Figure 5:**
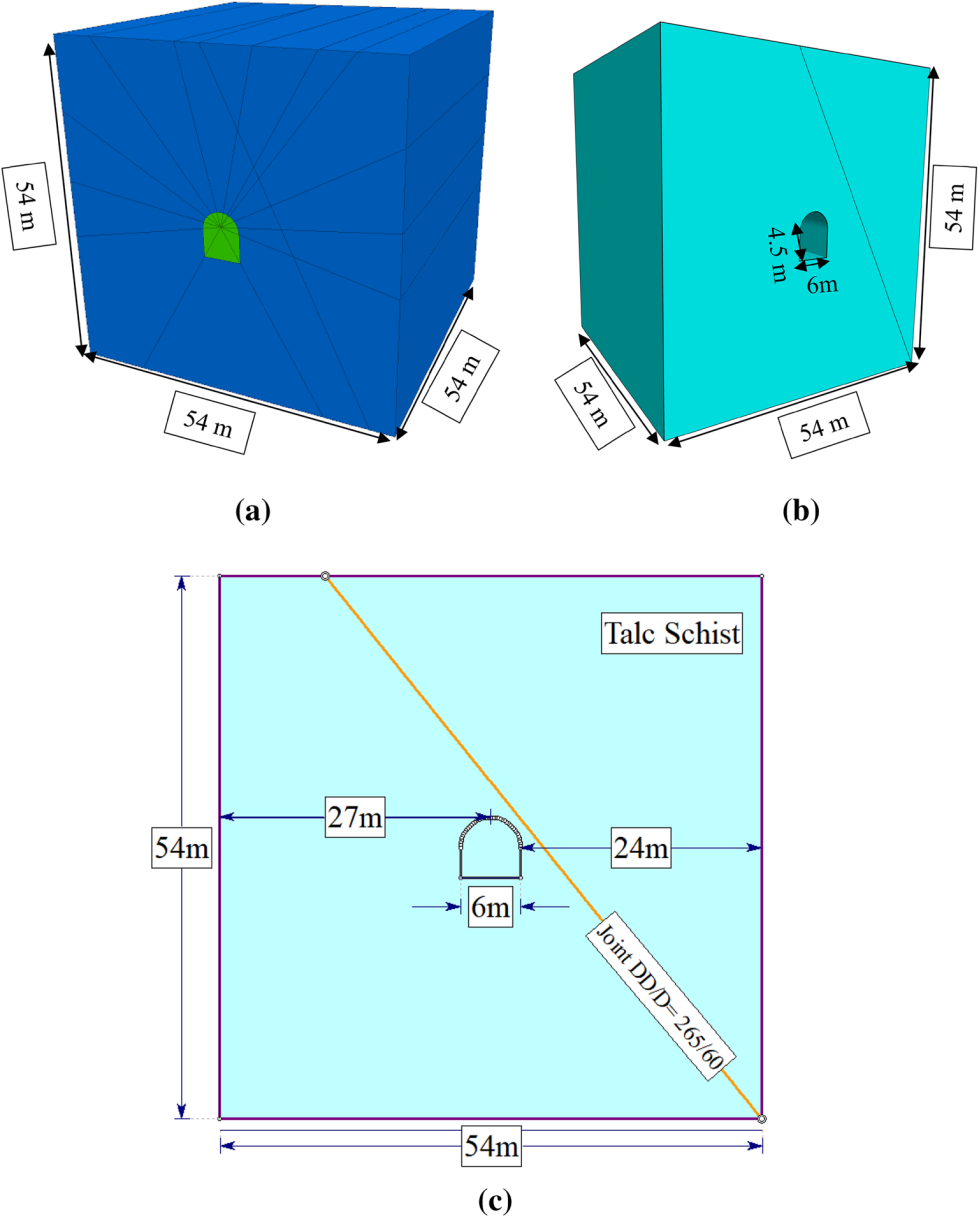
The geometries of 3DEC (**a**), FLAC3D (**b**) and RS2 (**c**).

To simulate the geometry and dimensions of the horseshoe tunnel in the Lega-Dembi gold mine, which had a width of 6 m, we employed a geometric model. The model accounted for the variation in the stress zone around the tunnel owing to the excavation. According to Saint–Venant’s principle, this zone typically ranges from three to five times the tunnel width from its boundary. Thus, we included a shield zone 24 m from the tunnel edge in the model, which enabled a comprehensive three-dimensional analysis. The model had the same measurements of 54 m × 54 m × 54 m in both 2D and 3D domains (Fig. 5). Additionally, the model incorporated a rock joint with a dip direction of 265° and dip angle of 60°, which exhibited failure.

#### Boundary condition and meshing

**Figure 6:**
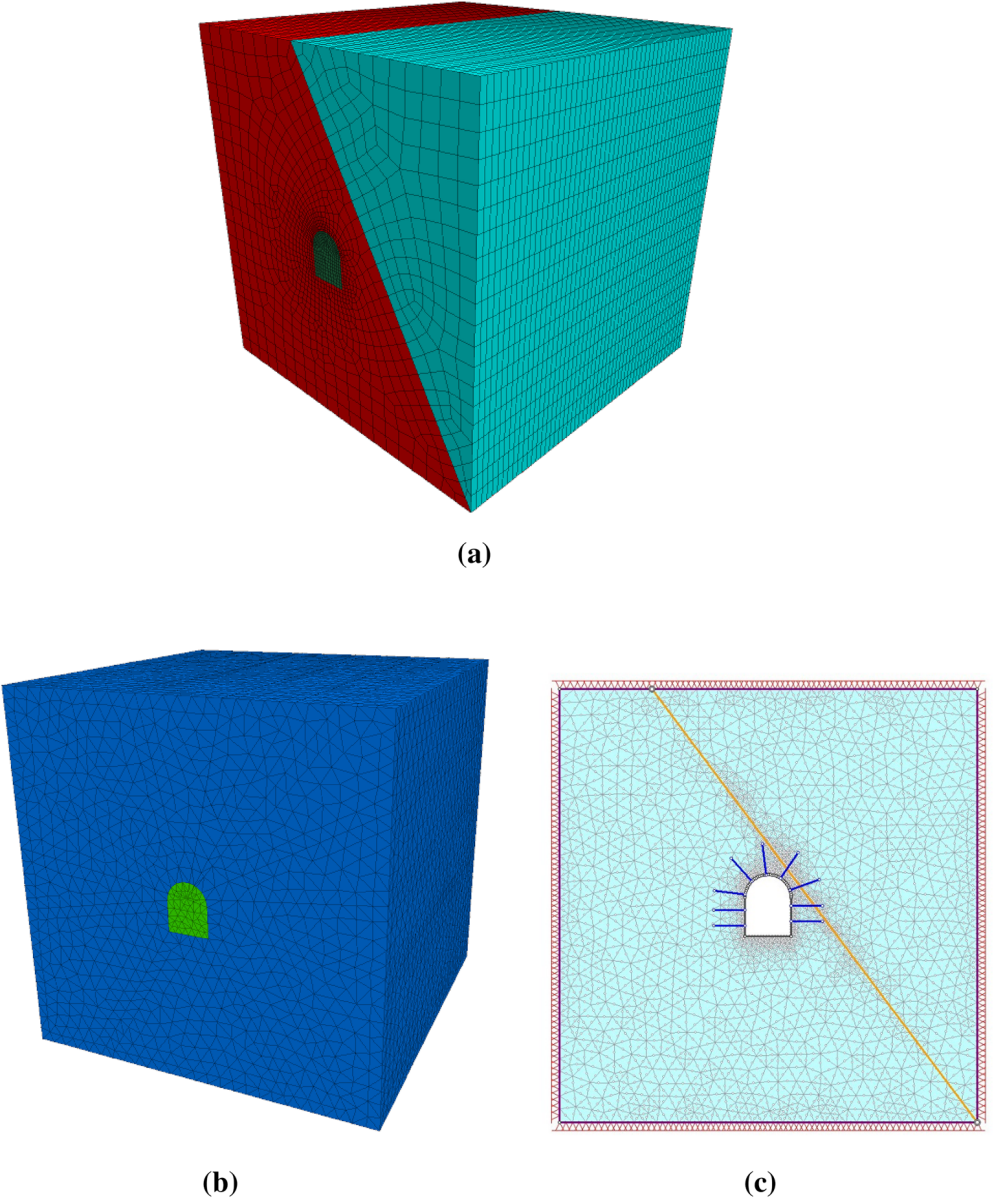
The FLAC3D (**a**), 3DEC (**b**), and RS2 (**c**) models with their boundaries and meshes.

The boundary conditions of the model were established based on the tunnel depth and radius. Messina et al.^32^ stated that a tunnel was considered deep if the depth-to-radius ratio exceeded 25. The tunnel had a ratio of more than 25, indicating that it was deep. Fixed boundary conditions were applied in all directions of the model. After setting all the boundaries, a mesh for the model was generated. For 3D modeling, tetrahedral zoning was employed, which could more effectively mesh irregular block shapes^33^ (Fig. 6a and b). For the 2D modeling, a six-node triangular element was used (Fig. 6c). A gravity stress field that varied linearly with depth from a user-defined ground surface elevation was established using the gravity field stress option.

#### Constitutive material model

The deformation of the rock mass was modeled using the continuum method with finite elements and finite difference methods. The generalized Hoek–Brown failure criteria were applied using the FLAC3D and RS2 software tools. These results were in contrast with those of the Distinct Element Code (3DEC), which employed the Mohr–Coulomb failure criterion.

#### Excavation and ground support

**Figure 7:**
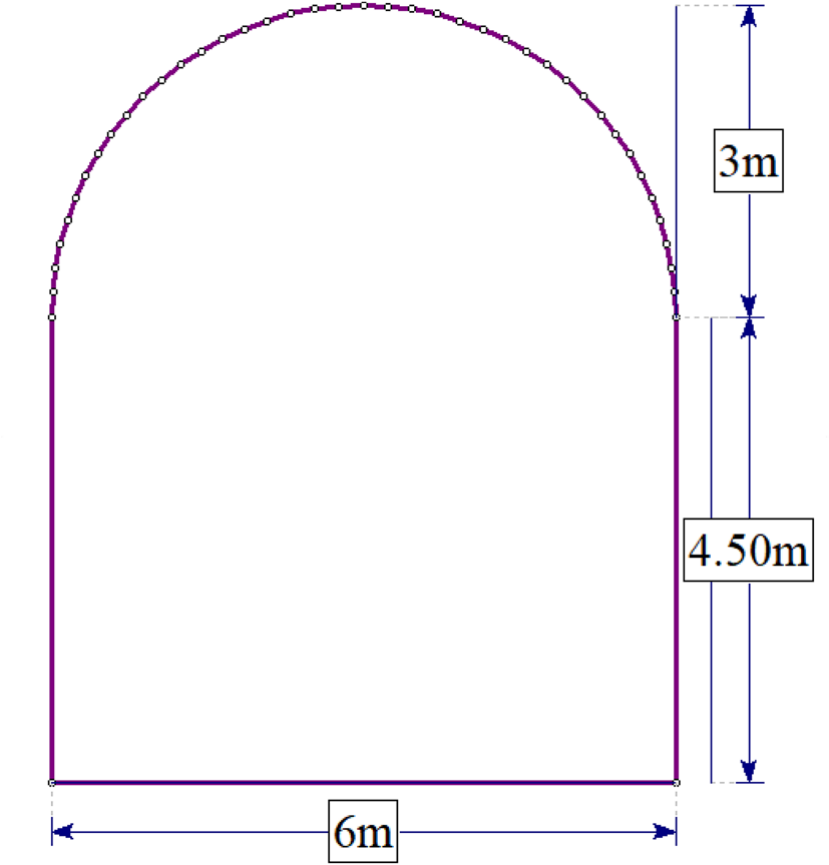
Shape and dimensions of the tunnel excavation.

The Midroc Lega-Dembi underground design manual, which stipulates the excavation procedures for various rock types, guides the tunnel excavation method. The rock class in the study area requires a full-face excavation method. This method excavates the entire cross-section of the tunnel simultaneously without any provisional openings or intermediate supports. The numerical model employed this method to simulate field conditions. Figure 7 illustrates the horseshoe shape of the tunnel, which has a width of 6 m and a height of 7.5 m.

#### Rock bolts properties

**Figure 8:**
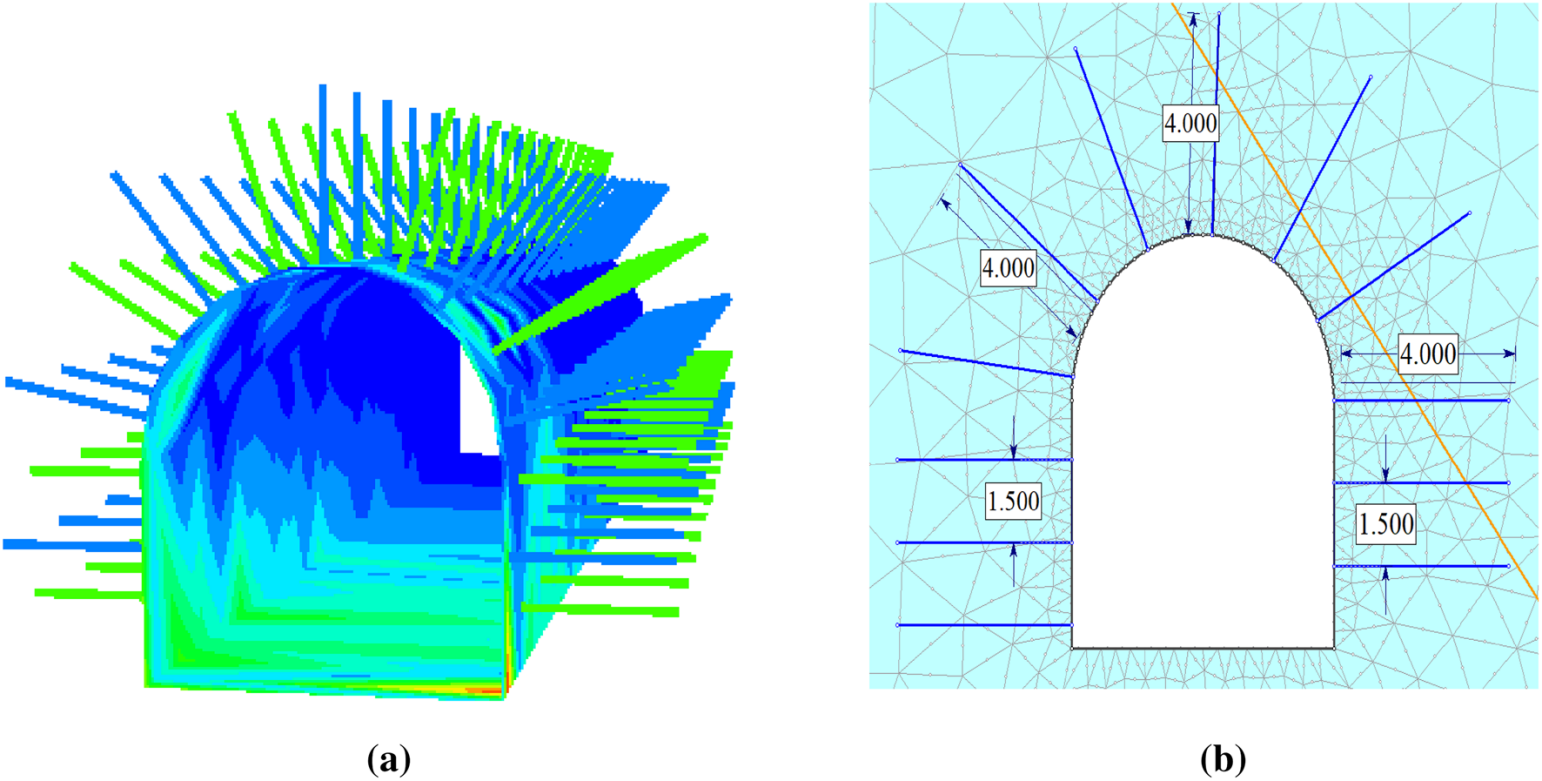
Rock bolt FLAC3D (**a**) and RS2 (**b**).

Swellex rock bolts, which create friction and mechanical interlocking with rock masses, were employed underground in the Lega-Dembi. Table 4 presents some Swellex bolts from Atlas Copco. The swellex bolts are simple to install and offer corrosion protection. They could also be lengthened by one segment to reach 4 m (Fig. 8).

**Table 4:** Properties of the rock bolt structure.

Support type	Diameter (mm)	Young’s modulus (GPa)	Ultimate yield load (kN)	KBond (MN/m/m)	Sbond (kN/m)
Rock Bolt	44	200	230	200	300

#### Shotcrete properties

The Midroc Lega-Dembi method of statement for shotcrete prescribes the use of shotcrete reinforced with steel fibers. The shotcrete has a characteristic compressive strength (fck) of 30 MPa. It was applied according to the specific indications for each support class and excavation section. The properties of shotcrete, such as Young’s modulus (E) and Poisson’s ratio, were estimated using the correlation provided by the reinforced concrete class C30. Table 5 lists the properties of the shotcrete.

**Table 5:** Shotcrete properties.

Support type	Thickness (mm)		Young’s modulus (E)	Poisson’s ratio
	Initial lining	Final lining	(GPa)	
Shotcrete	100	100	32	0.25

#### Analysis of plastic zone of tunnel surrounding rock

**Figure 9:**
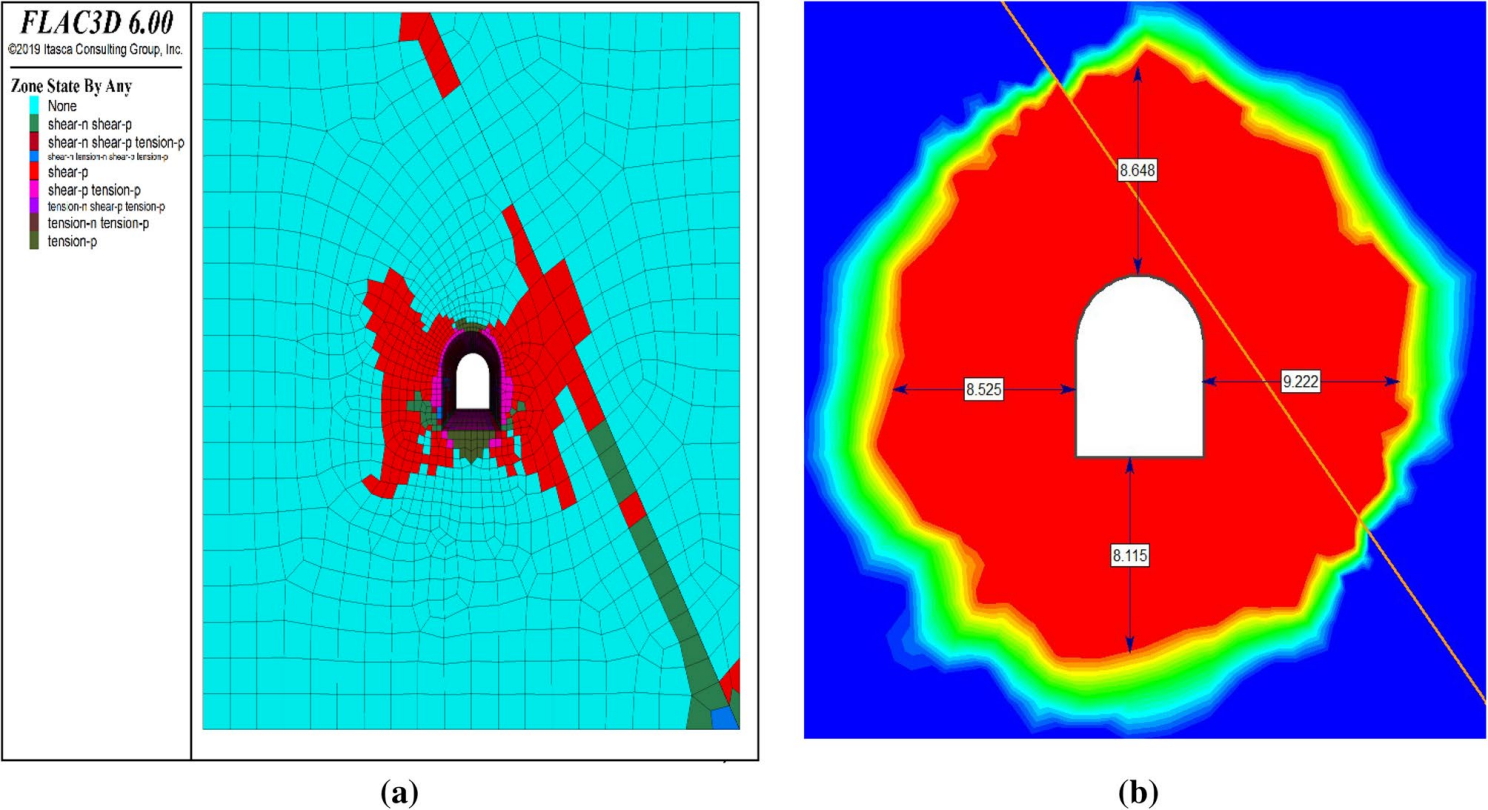
Plasticity zone (**a**) FLAC3D and (**b**) RS2.

The plastic zone is the region of rock mass around the tunnel that undergoes irreversible deformation due to the excavation-induced stress redistribution. The plasticity zone can affect the stability and performance of the tunnel and its support system (Fig. 9). In summary, the large deformation control measures of the tunnel are active reinforcement, stratified support, combined long and short, reduced disturbance, and reserved deformation.

#### Parametric study

This study examined the effects of various parameters on the tunnel deformation. The factors considered in the study were the geotechnical parameters (GSI, UCS, E, and D), tunnel size, rock joint properties, and support systems. This study assessed the stability of a tunnel based on these factors and compared the results with field data and measurements. Furthermore, this study investigated how the concrete lining material influenced the tunnel squeezing behavior.

### Numerical validation

#### Model validation by using analytical method

**Figure 10:**
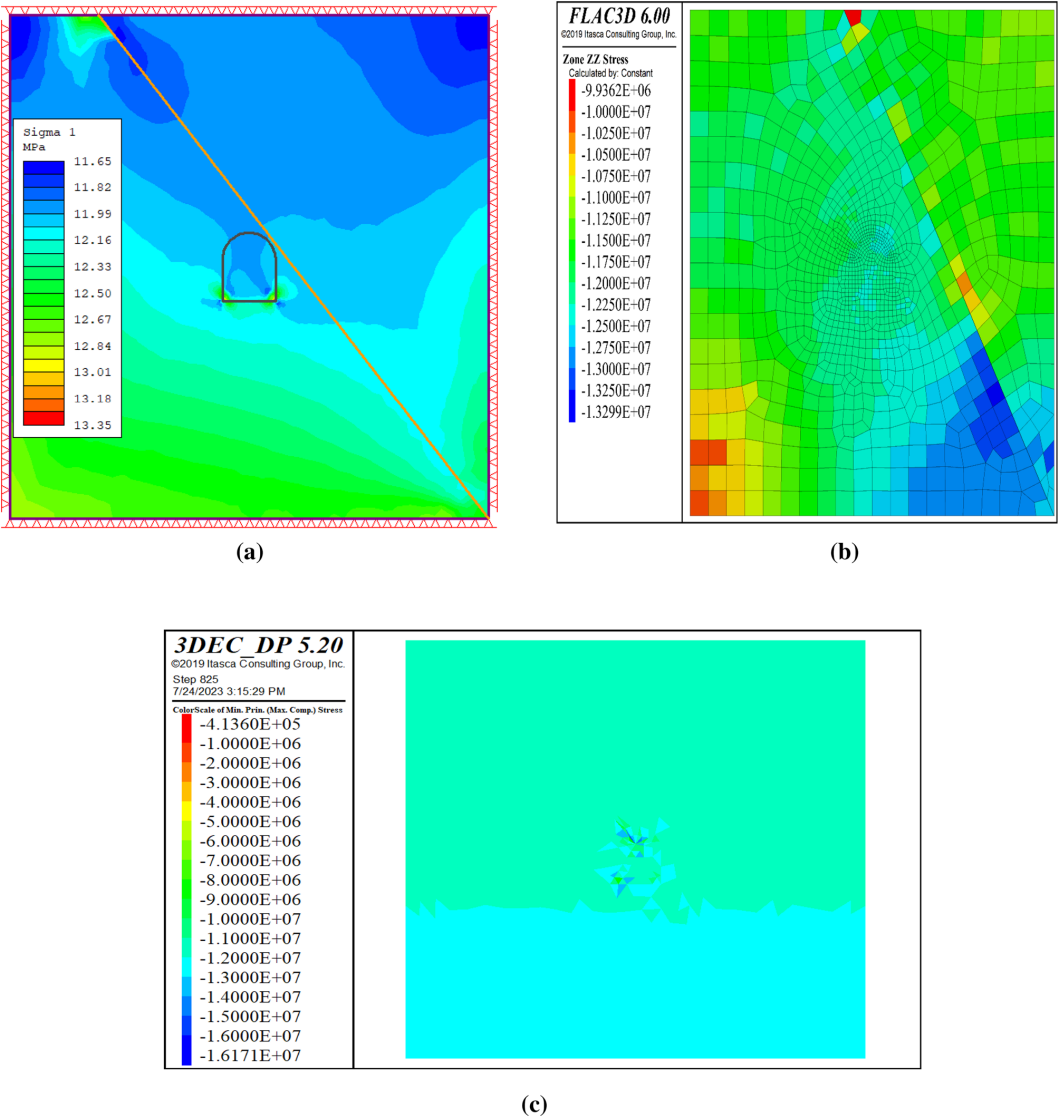
Stress contours by RS2 (**a**), FLAC3D (**b**), and (**c**) 3DEC for validation.

This section assesses the validity of the proposed 2D and 3D numerical approaches for the tunnel stability analysis. The numerical model was verified using the in-situ stress of the tunnel rock mass. The vertical overburden stress of the rock mass was derived from the average unit weight of the overlying material (26.80 kN/m^3^) and tunnel depth (440 m) in the study section. The vertical stress at the top of the tunnel is 11.792 MPa. Figure 10 shows the numerical results for the continuum and discontinuum approaches. The contour plots demonstrate that the in-situ stresses at the top of the tunnel were 11.99 MPa for the continuum approach and slightly above 11.99 MPa for the discontinuum approach. These results concur with manual calculations, particularly for the continuum approach. Hence, the conclusion relied on the continuum approach, and the discontinuum approach was used only for comparison.

#### Model validation

This study employed FLAC3D software to re-evaluate the results of Yu et al.^34^, who examined the Da Pingshan Tunnel in a karst cave environment using the Phase II software. The tunnel has a horseshoe-shaped cross-section situated near the karst region of China. A 6 m diameter horseshoe karst cave was modeled with the parameters presented in Table 6, and the effect of the distance between the tunnel and cave on the tunnel displacement was analyzed. These findings confirmed the conclusion of Yu et al.^34^ that the displacement diminished as the distance increased. The model boundaries were fixed to restrict movement in both horizontal and vertical directions. The karst cave was vacant, and the excavation was initiated by drilling and lining the tunnel. Table 7 lists the lining specifications. The model dimensions were 150 × 40 × 72 m, and the mesh densities varied near the tunnel.

**Table 6:** Characteristics of the Da Pingshan tunnel used for modeling by Yu et al.^34^.

Young’s modulus (GPa)	Poisson’s ration	Specific weight (kN/m^3^)	Cohesion (GPa)	Internal friction
5	0.30	23	0.5	35

**Table 7:** The characteristics of the tunnel lining reported by Yu et al.^34^.

Poisson ratio	Young’s modulus (GPa)	Thickness (m)
0.28	20	0.20

**Figure 11:**
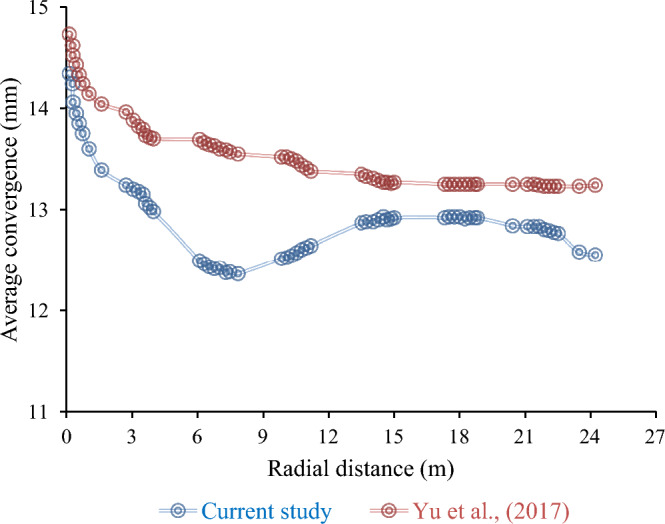
Comparative analysis between the findings of Yu et al.^34^ and the current study.

As Fig. 11 illustrates, the results of Yu et al.^34^ align with those of our research on convergence edge changes. However, the model outcomes in this study are marginally lower than those reported by Yu et al.^34^. This difference might have resulted from the absence of integer stress relaxation values in the RS2 software. Nonetheless, this variation did not alter the overall consistency between our validation model and the results of Yu et al.^34^, confirming our findings.

## Results and discussion

**Figure 12:**
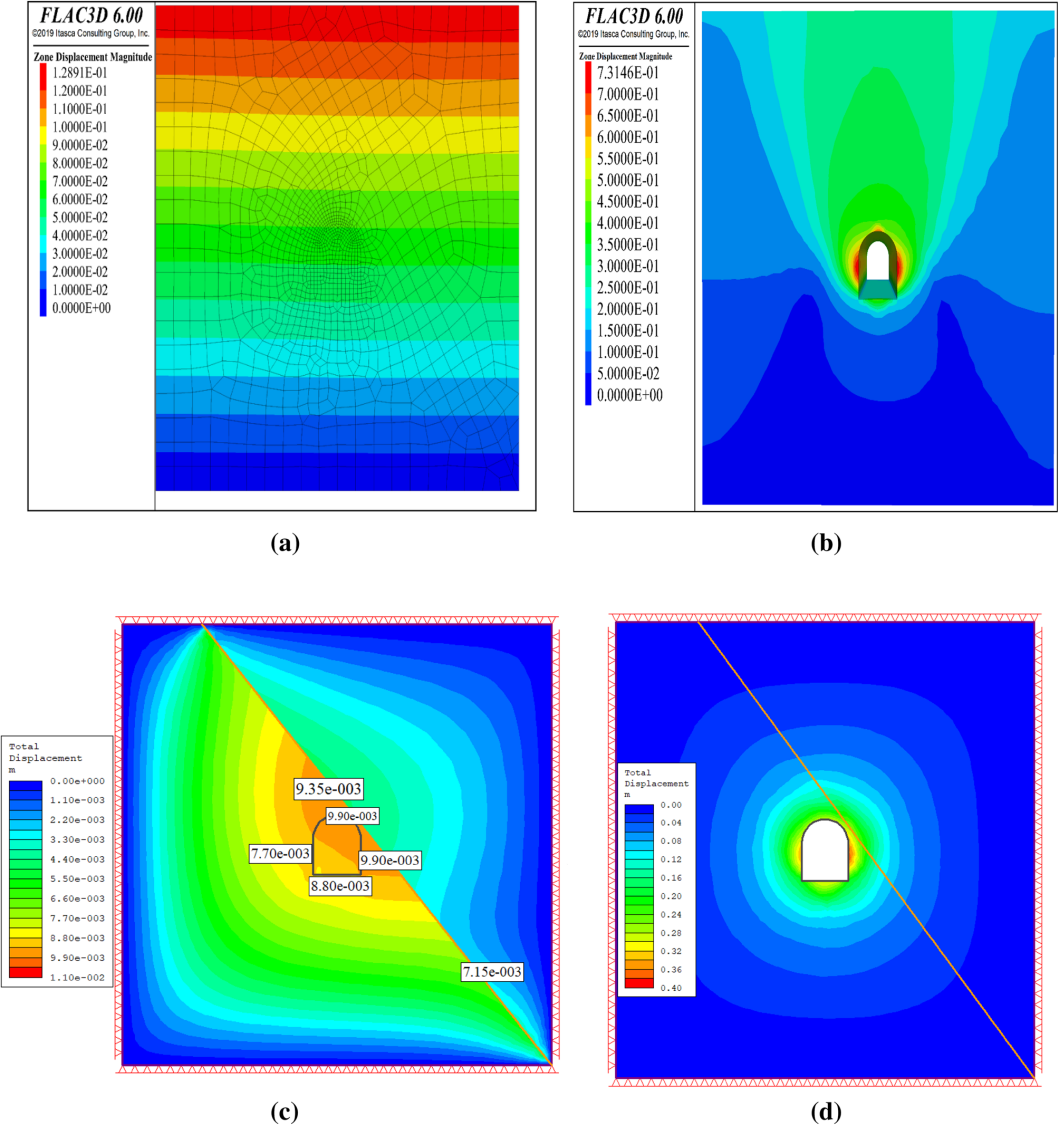
(**a**) In-situ stage total displacement contour by FLAC3D, (**b**) Excavation stage total displacement contour by FLAC3D, (**c**) In-situ stage total displacement by RS2, (**d**) Excavation stage total displacement contour by RS2.

The study used the tunnel design parameters in section “Parametric study using continuum modeling” for numerical modeling. Three stages were simulated: in-situ, excavation, and support systems. Figure 12 shows the total displacement contours for each stage. The initial stage exhibits the highest displacement contour near the joint structure (Fig. 12). The displacement contour did not improve after the tunnel excavation (Fig. 12). This indicates that the tunnel-supporting mechanism failed to reduce tunnel squeezing deformation.

**Figure 13:**
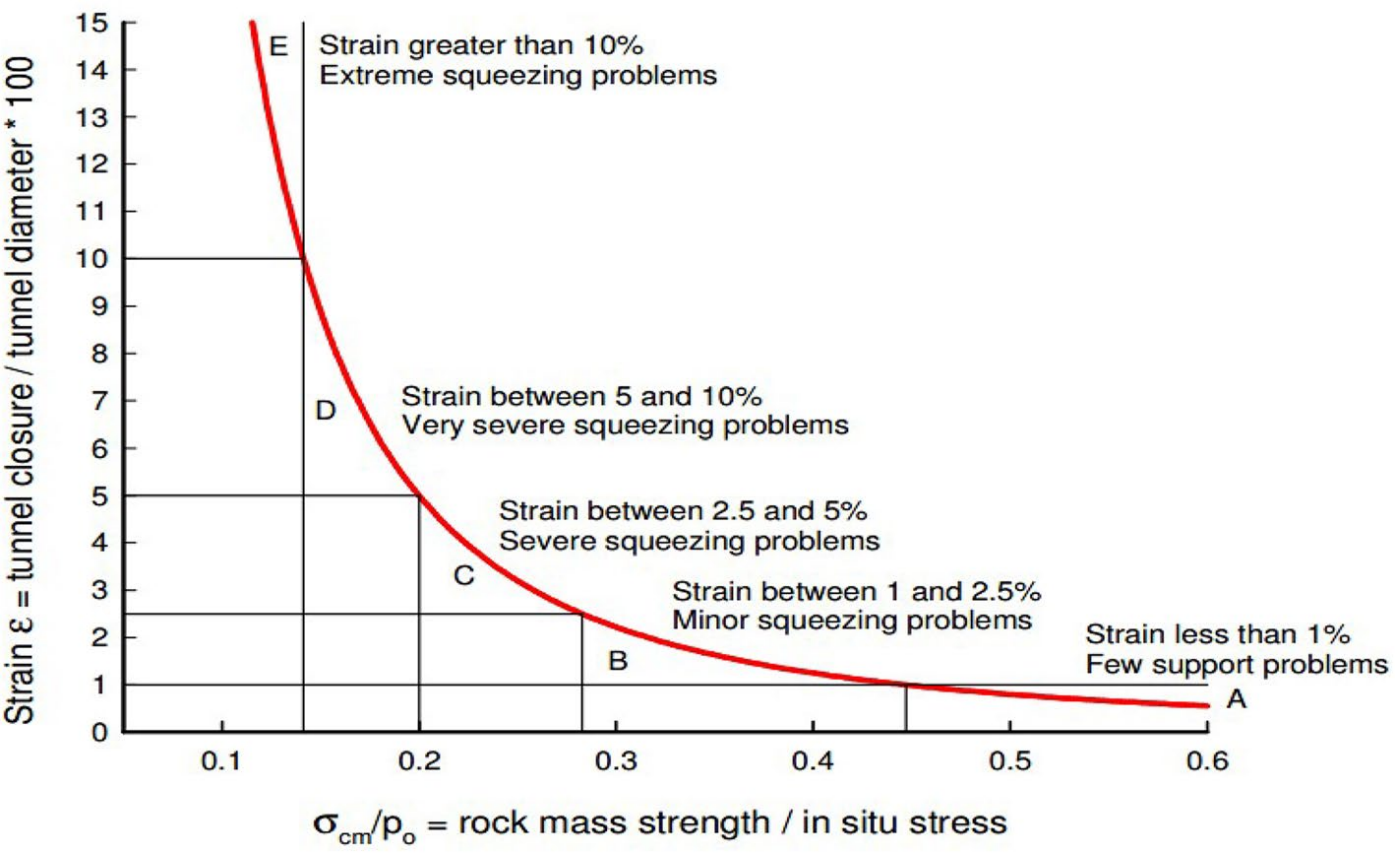
Classification of squeezing behavior^35^.

**Figure 14:**
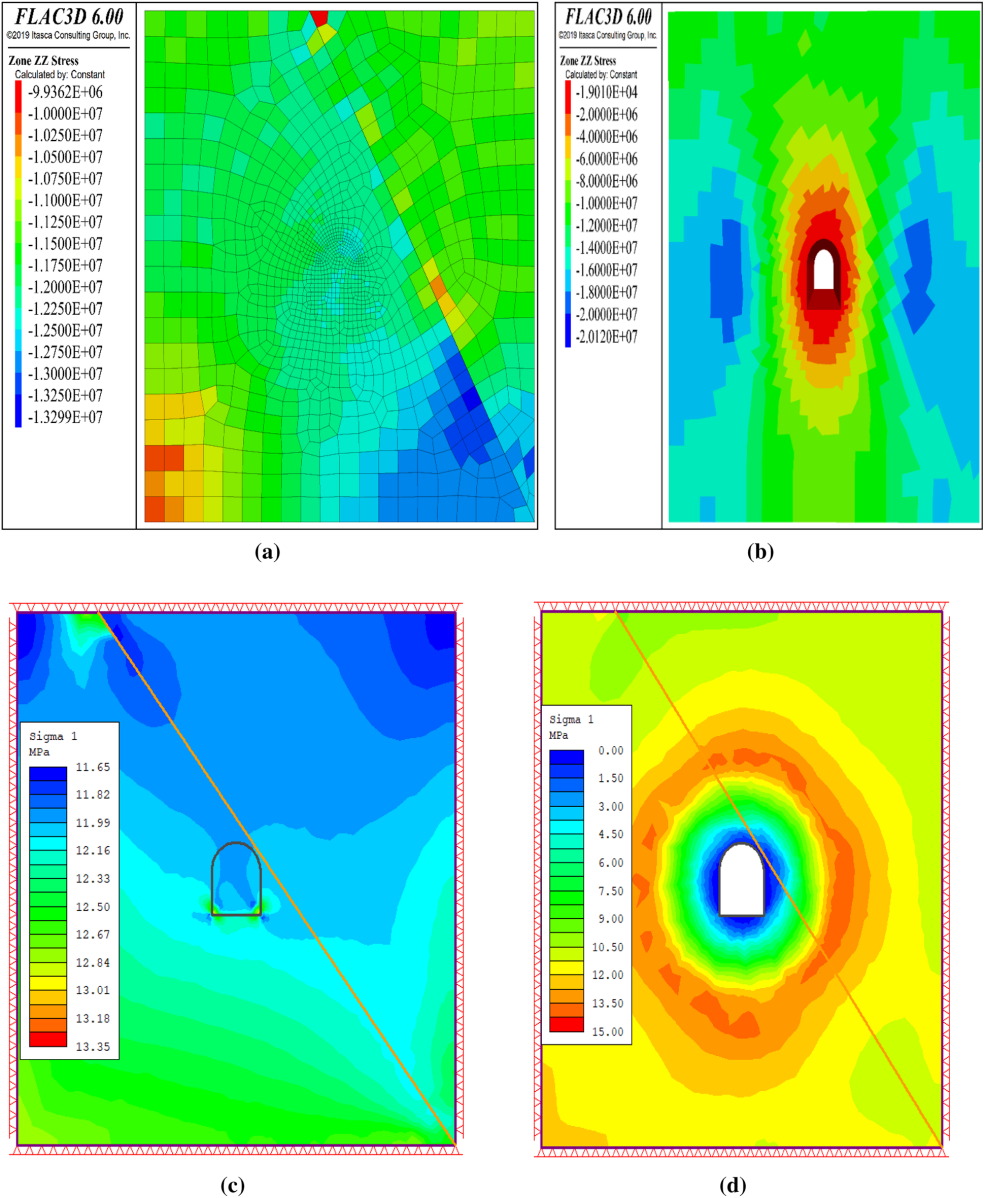
(**a**) In-situ principal stress contour by FLAC3D, (**b**) Excavation principal stress contour by FLAC3D, (**c**) In-situ principal stress contour by RS2, (**d**) Excavation principal stress contour by RS2.

The study classified the tunnel as category D in Fig. 13 using Hoek^35^ curves, indicating a severe squeezing problem. This category was based on tunnel strain, which was 5.84% of the ratio of the maximum displacement (0.35 m) to the tunnel radius (6 m). Hoek^35^ recommended strong supports, such as concrete linings and light steel; however, this tunnel only had rock bolts. This implies that the tunnel failure was partly due to insufficient support systems. Figure 14 illustrates that the tunnel excavation altered the in-situ stress distribution and generated a maximum principal stress contour near the tunnel walls. This demonstrates that the tunnel excavation affected both the magnitude and location of the principal stress (Fig. 14).

### Parametric study using continuum modeling

#### Influence of a supported and unsupported tunnel on total displacement

**Figure 15:**
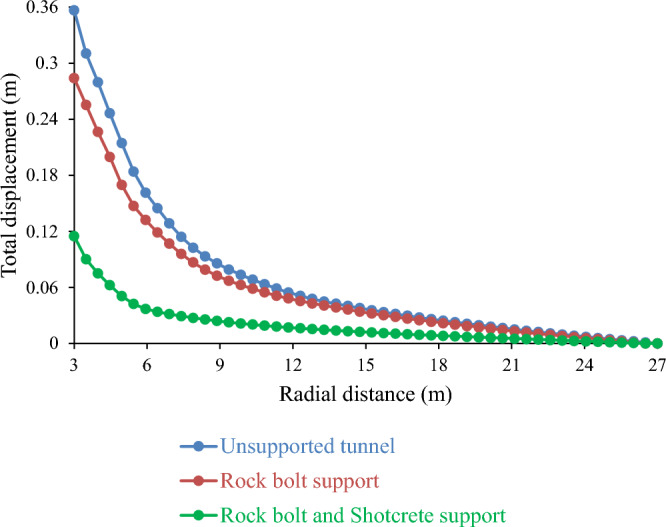
Effect of deformation in supported and unsupported tunnels on total displacement.

Rock support systems are essential for ensuring the safety and stability of mining tunnels, particularly under difficult geological conditions. Figure 15 shows the effects of different types of rock support systems on the total displacement of the tunnel. The results revealed that the unsupported tunnels had the highest total displacement (0.36 m), suggesting a high risk of rockfalls and cave-ins. In contrast, supported tunnels have a much lower total displacement, depending on the type of support used. For instance, using only rock bolts as support reduces the total displacement to 0.28 m, which is a 22.22% improvement over the unsupported case. However, using both rock bolts and shotcrete as support decreases the total displacement even further to 0.11 m, which is a remarkable 69.44% improvement over the unsupported case and a 60.71% improvement over the rock bolt-only case. These results demonstrate the significant benefits of using rock support systems to enhance tunnel stability and mining efficiency. They also validated previous studies reporting similar results^24,36^.

#### Influence of excavation size on total displacement

**Figure 16:**
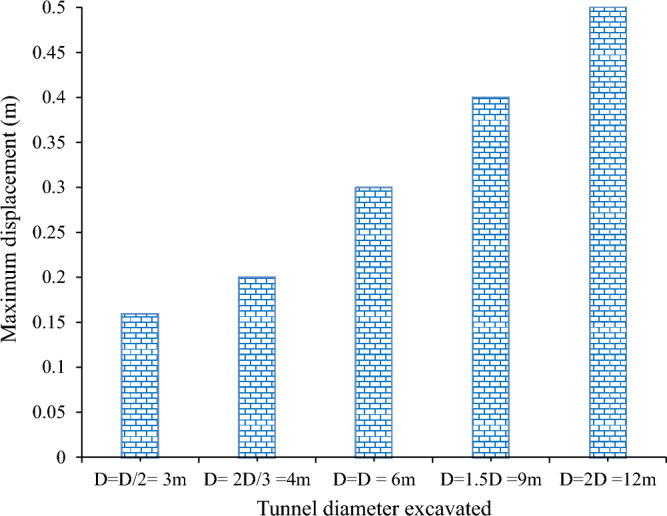
Effect of excavation dimension on total displacement.

This study examined the effect of the tunnel size on the deformation of the surrounding rock mass. The tunnel diameter ranged from 3 to 12 m, while other parameters, such as the overburden, rock properties, and support system, were constant. The study found that the rock mass displacement increased with the tunnel diameter but not linearly. For example, when the tunnel diameter increased from 6 to 12 m, the rock mass displacement increased by 40%. However, when the tunnel diameter decreased from 6 to 3 m, the rock mass displacement decreased by 53%. Figure 16 presents the results of the graph. This study validates previous research that also reported a positive correlation between the tunnel diameter and rock mass displacement^37,38^.

#### Effect of geotechnical parameters on total displacement

##### Effect of Geological Strength Index (GSI) on tunnel deformation

**Figure 17:**
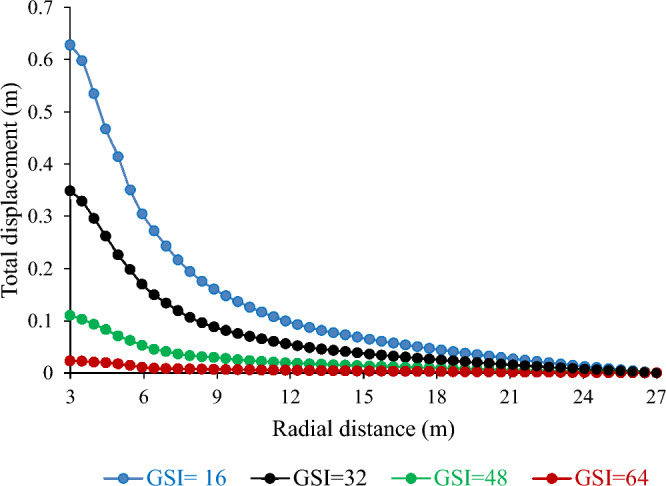
Effect of geological strength index on total displacement.

The quality of rock mass (GSI) is vital for geotechnical engineering. It affects the strength and deformation of the rock mass, which influences the stability and behavior of underground excavations, such as mining tunnels. This study investigated the effect of the GSI on the total displacement of the tunnel. Figure 17 shows that the total displacement varied inversely with GSI. For example, a 50% decrease in the GSI resulted in an 80.17% increase in the total displacement. Conversely, a 33.33% and 50% increase in the GSI led to a 68.39% and 93.39% decrease in the total displacement, respectively. These results suggest that the GSI has a considerable impact on tunnel stability and performance. A higher GSI implies a lower total displacement and vice versa. These results are in line with those of previous studies that reported a similar relationship between the GSI and total displacement^39–41^.

##### Effect of Unconfined Compressive Stress (UCS) on total displacement

**Figure 18:**
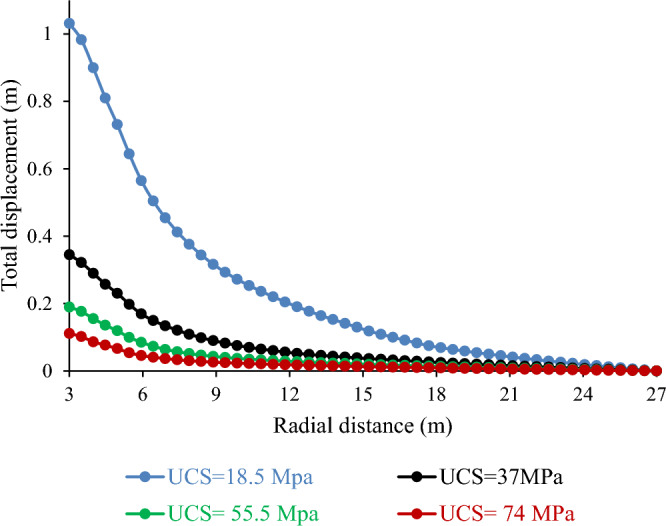
Effect of unconfined compressive stress on total displacement.

The strength of a rock mass (UCS) is vital for tunnel stability and convergence. This affects the deformation and failure of the rock mass, which influences the movement of the tunnel wall. Figure 18 shows that the total displacement varies inversely with the UCS. For example, a 50% decrease in the UCS resulted in a 99.85% increase in the total displacement. Conversely, a 33% and 50% increase in the UCS led to a 45.05% and 67.73% decrease in the total displacement, respectively. These results suggest that the UCS has a considerable impact on tunnel stability and performance. A higher UCS implies a lower total displacement and vice versa. They also validated previous studies, which reported similar results^42–44^.

##### Influence of Young’s modulus (E) on tunnel deformation

**Figure 19:**
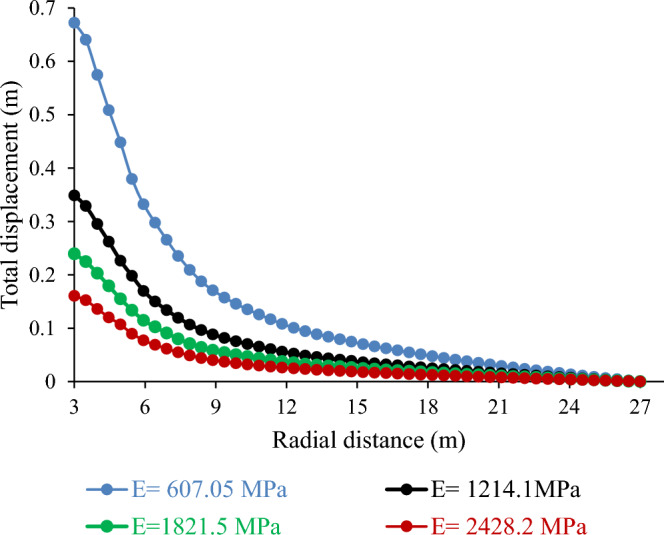
Effect of Young’s modulus on total displacement.

The stiffness of the rock mass (E) is vital for tunnel deformation during mining. This affects the deformation and failure of the rock mass, which influences the movement of the tunnel wall. Figure 19 demonstrates that the total displacement varies inversely with E. For instance, a 50% decrease in E resulted in a 93.10% increase in the total displacement. Conversely, a 33.34% or 50% increase in E led to a 45.60% or 33.05% decrease in total displacement, respectively. These results suggest that E has a considerable impact on the tunnel stability and performance. A higher E implies a lower total displacement and vice versa. These results are in line with those of previous studies that reported a similar relationship between E and the total displacement^37,45^.

##### Influence of Disturbance factor (D) on tunnel deformation

**Figure 20:**
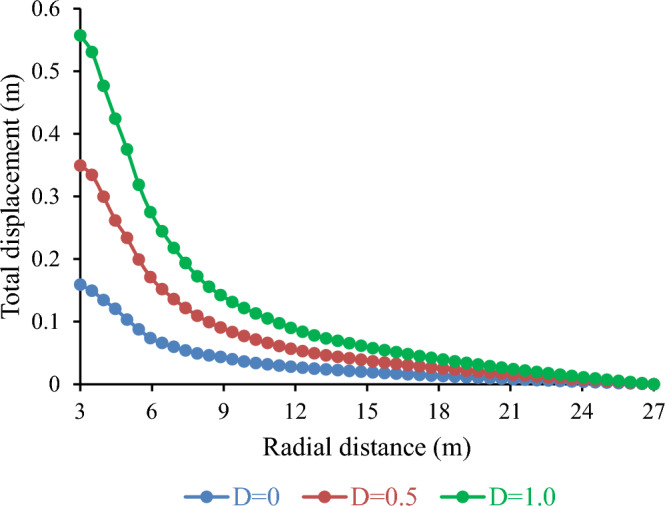
Effect of disturbance factor (D) on total displacement.

In this study, the effects of disturbance factors on the total displacement of the tunnel were investigated. The disturbance factor represents the damage and stress relief in the rock mass due to the excavation method. The total displacement is the radial movement of the tunnel wall caused by rock mass deformation and failure. Figure 20 shows that the total displacement increased with the disturbance factor. For example, a 50% decrease in the disturbance factor resulted in a 54.48% decrease in the total displacement. Conversely, a 50% increase in the disturbance factor resulted in a 59.49% increase in the total displacement. These results suggest that the disturbance factor has a considerable impact on the tunnel deformation and stability. These results are in line with those of previous studies, which reported a linear relationship between the disturbance factor and the total displacement^46–48^.

#### Influence of rock joint on total displacement

**Figure 21:**
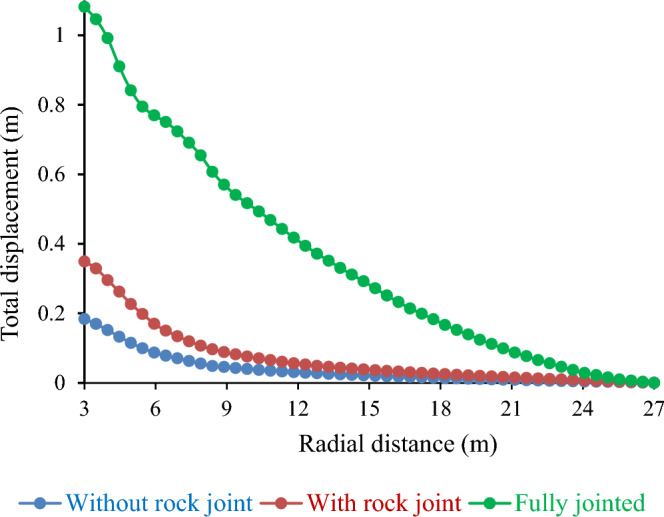
Influence of rock joint on total displacement.

In this study, the effects of rock joints on the total displacement of the tunnel were investigated. Rock joints are discontinuities in a rock mass that can cause various tunnel instability and fracture problems. They also disturb the stress distribution and generate additional stress in the rock mass. These challenges affect ground support systems in mining tunnels, as conventional measures may be inadequate. Figure 21 shows that the total displacement is the radial movement of the tunnel wall caused by rock mass deformation and failure. The figure shows that the total displacement increased with the number of rock joints in the tunnel. For instance, a tunnel with no rock joints has a total displacement of 0.18 m, while a tunnel with fully rock joints has a total displacement of 1.08 m, which is a 142.85% increase. These results suggest that rock joints have a detrimental effect on tunnel stability and increase the risk of tunnel collapse. These results are in line with those of previous studies that reported a significant impact of rock joints on tunnel stability^49,50^.

In summary, Table 8 presents an overview of the geotechnical parameters and emphasizes the highest value for each parameter.

**Table 8:** Effect of geotechnical parameters on total displacement.

GSI	16	32	48	64
	0.627	0.348	0.110	0.023
UCS	18.5 MPa	37 MPa	55.5 MPa	74 MPa
	1.031	0.344	0.189	0.111
E	607.5 MPa	1214.1 MPa	1821.5 MPa	2428.2 MPa
	0.672	0.348	0.239	0.160
D	0	0.5	1.0	
	0.159	0.349	0.557	
Rock joint	Without rock joint	With rock joint	Fully jointed	
	0.183	0.348	1.081	

### Discontinuous modeling results

#### In-situ and induced stress distribution

**Figure 22:**
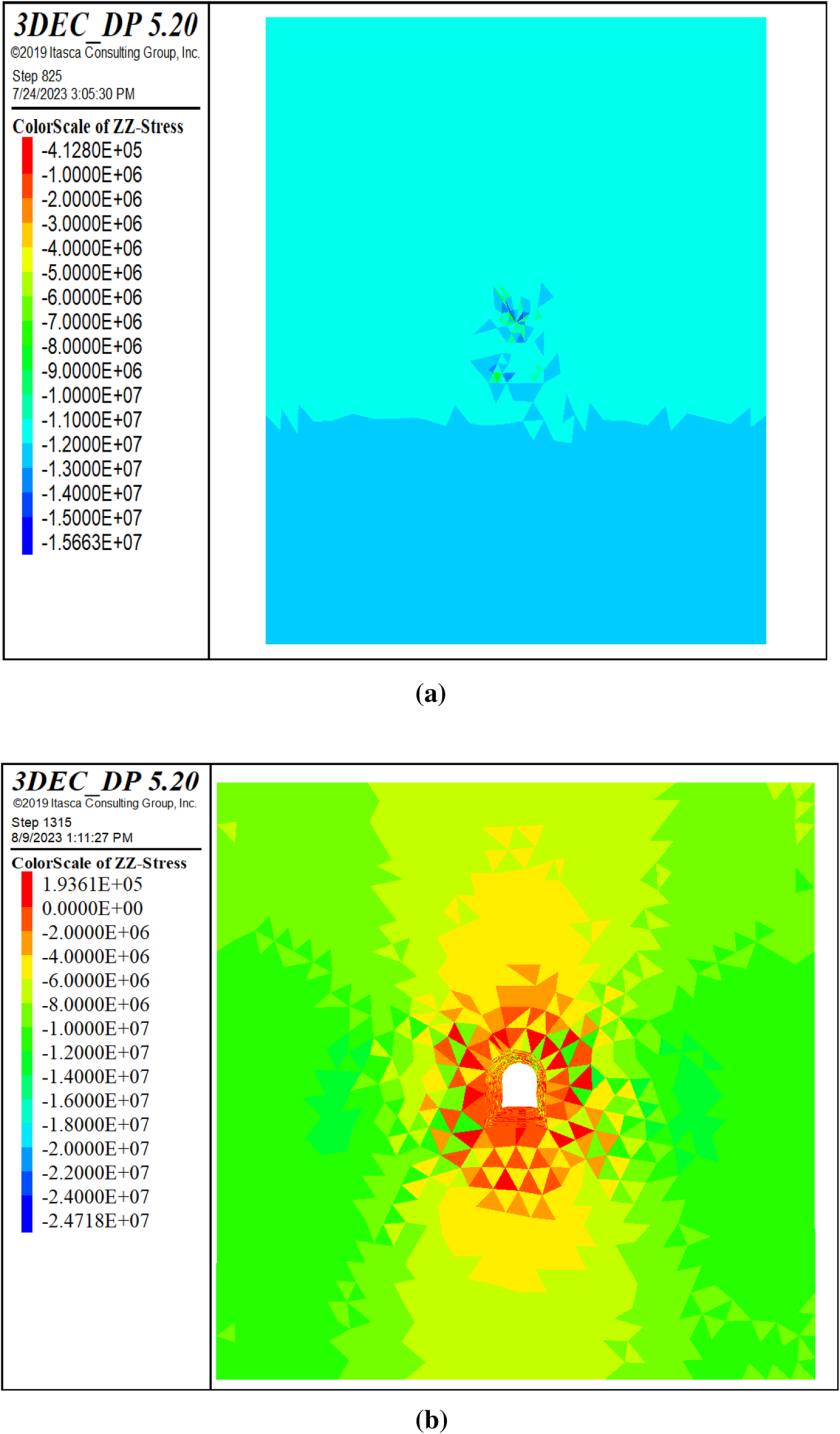
Variation of vertical stress before excavation (**a**) and after excavation (**b**).

This section presents the results of stress analysis following the simulation of the excavation process. Figure 22 illustrates the stress distribution of the maximum principal stress around the tunnel before and after excavation. The excavation process increased the compressive stress around the tunnel from 16.171 to 27.216 MPa (compression). The induced stress, which is the additional compressive stress owing to the excavation process, was 11.045 MPa (compression), as reported in^11^. This implies that the tunnel underwent an additional compressive stress of 11.045 MPa owing to the excavation process.

#### Deformation

**Figure 23:**
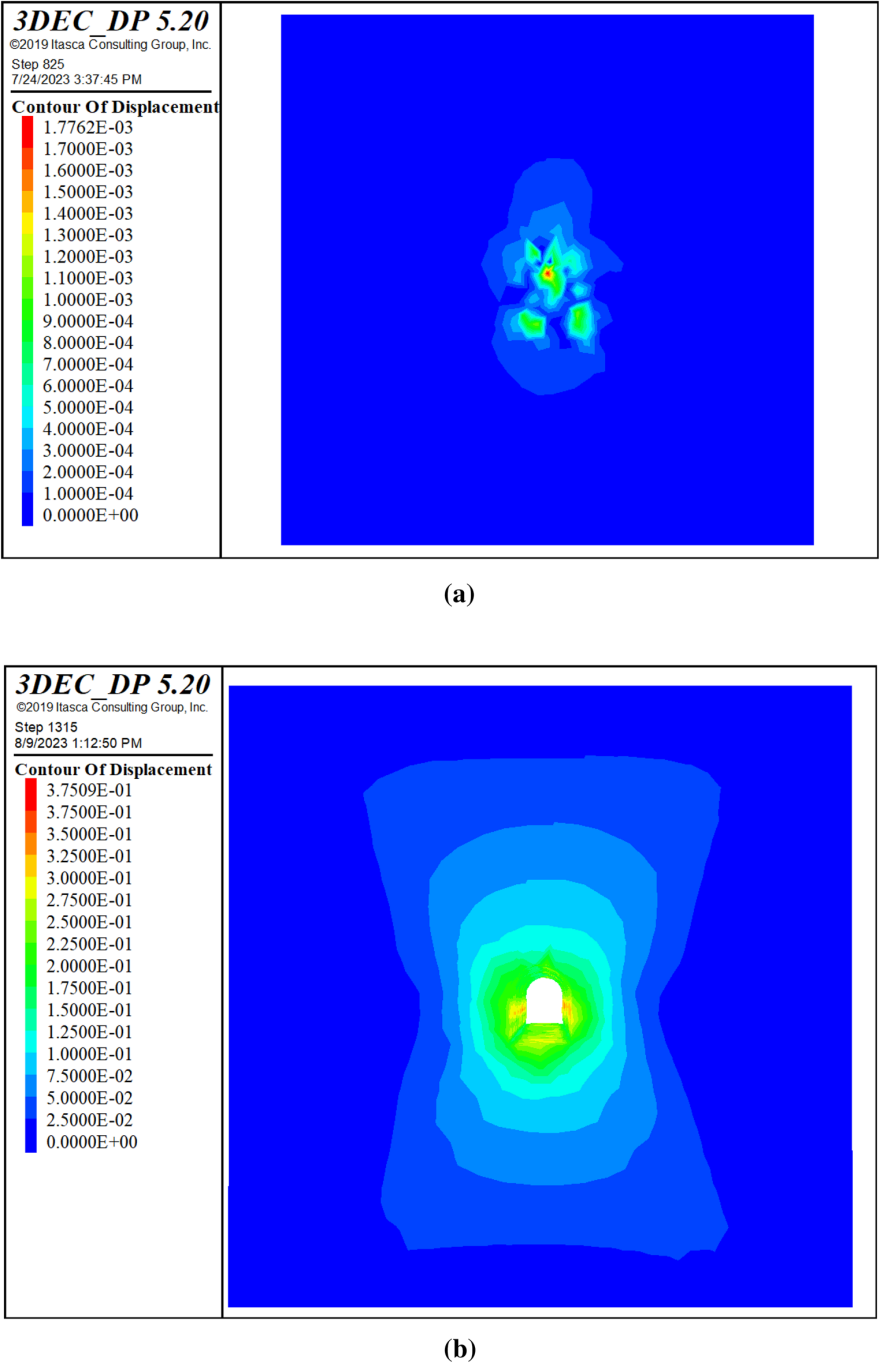
Total displacement contours before excavation (**a**) and after excavation (**b**).

This section presents the results of deformation analysis following the simulation of the excavation process. Figure 23 shows the contour plot of the total displacement, which reached a maximum value of 0.375 m. The figure demonstrates that the displacement is greatest at the tunnel boundaries, particularly at the top and bottom, where the rock mass experiences the highest stress concentration. The displacement magnitude decreased as the distance from the tunnel boundary increased and became zero at the end of the model. This suggests that tunnel excavation has a negligible effect on the rock mass far-field deformation.

#### Contrast between continuum and discontinuum methods

This study investigated the mechanical behavior of rock masses using the continuum method. However, some studies applied the discontinuum method for comparison. This section compares the results of both methods in terms of deformation and stress based on the maximum values obtained from numerical simulations. The comparison highlights the advantages and disadvantages of each method and provides insights into the appropriateness of the continuum method for modeling complex rock-mass problems. Table 9 presents a comparison of the continuum and discontinuum methods for numerical modeling of rock masses. The results showed that both methods produced similar stress distribution patterns; however, the displacements were smaller in the discontinuum method. This difference may be explained by the variations in the input data and constitutive models used for each method.

**Table 9:** Contrast between continuum and discontinuum methods.

Parameter	Unit	Continuum approach (RS2)	Continuum approach (FLAC3D)	Discontinuum approach (3DEC)
Initial stress	MPa	11.99	12.5	13
Post excavation stress	MPa	15	20.12	25
Initial displacement	m	0.00935	0.05	0.001776
Final displacement	m	0.40	0.731	0.375

## Conclusions

In this study, various numerical modeling techniques were applied to assess the collapse of the Lens-2 tunnel in the Lega-Dembi underground gold mine. The tunnel behavior was simulated using 2D and 3D finite element methods (RS2 and FLAC3D, respectively), which considered a continuous rock mass. A parametric analysis was conducted using the same methods to examine how different factors such as rock mass properties and support systems affect tunnel stability. The results of the continuous numerical models were then compared with those of the discontinuous numerical models, which were obtained using the three-dimensional distinct element method (3DEC). Discontinuous numerical models can account for the effects of rock-mass discontinuities, such as joints and faults, on tunnel performance.

Based on these findings, this study comes to the following conclusions were drawn:

1. This study evaluated the tunnel behavior and the convergence in the compressive rock mass using numerical simulations. The right wall of the tunnel exhibited the largest displacement, which was 0.40 m, and it reduced as it moved away from the boundary. The tunnel had a convergence of 5.84%, which required strong support systems, such as rock bolts and concrete lining. However, the rock bolts caused some failures, suggesting the need for alternative support methods. This study provides valuable insights into tunnel construction and design in a geological setting.
2. The parametric analysis demonstrated that the combination of rock bolt and shotcrete support elements considerably enhanced the stability of the underground excavations, decreasing the total displacement by 69.44%. Hence, we suggest using 4 m long rock bolts and shotcrete for this mining site. Furthermore, the spacing and pattern of rock bolts should be adjusted to guarantee sufficient coverage and reinforcement of the rock mass. This would offer a complete and dependable support system for the underground excavations.
3. The displacement criterion defines the stability of the rock mass around the tunnel, which relies on several key factors. This study conducted a sensitivity analysis to examine the impact of these factors on the displacement criterion. These factors comprise the rock mass deformation and strength properties, tunnel dimensions, overburden, geometry, and joint.
4. This study examined the effect of geotechnical parameters on tunnel stability in a tunnel excavation project. The tunnel displacement was evaluated using parametric analysis and numerical simulations under varying values of the GSI and UCS. The results indicated that these parameters had a considerable influence on the tunnel performance. The tunnel displacement increased by 80.17% and 99.85% when the GSI and UCS decreased by 50%, respectively.
5. The stress and displacement distributions were evaluated using numerical modeling outcomes from the continuum and discontinuum methods. The outcomes showed that both methods produced comparable stress patterns; however, the displacement values were smaller when the discontinuum method was applied. This difference may have resulted from variations in the input data and constitutive models for each method.

This study establishes the basis for ongoing efforts to address the underground instability problem at the Midroc Lega-Dembi Gold Mine. This underscores the significance of a comprehensive approach that accounts for geological, geotechnical, and operational factors to ensure safe and sustainable mining practices.

## Data Availability

The corresponding author provides data that support the findings of this study upon reasonable request.
